# Comparison of [^3^H]-Thymidine, Carboxyfluorescein Diacetate Succinimidyl Ester and Ki-67 in Lymphocyte Proliferation

**DOI:** 10.3389/fped.2022.638549

**Published:** 2022-04-25

**Authors:** Hsin-Ju Lee, Chun-Chun Gau, Wan-Fang Lee, Wen-I Lee, Jing-Long Huang, Shih-Hsiang Chen, Ho-Yu Yeh, Chi-Jou Liang, Shih-Hang Fu

**Affiliations:** ^1^Division of Allergy, Asthma, and Rheumatology, Department of Pediatrics, Chang Gung Memorial Hospital, Chang Gung University College of Medicine, Taoyuan, Taiwan; ^2^Department of Pediatrics, Chang Gung Memorial Hospital, Keelung, Taiwan; ^3^Division of Pediatric General Medicine, Department of Pediatrics, Chang Gung Memorial Hospital and Chang Gung University College of Medicine, Taoyuan, Taiwan; ^4^Primary Immunodeficiency Care and Research (PICAR) Institute, Chang Gung Memorial Hospital, Chang Gung University College of Medicine, Taoyuan, Taiwan; ^5^Department of Pediatrics, New Taipei Municipal TuCheng Hospital, New Taipei City, Taiwan; ^6^Division of Hematology/Oncology, Department of Pediatrics, Chang Gung Memorial Hospital, Taoyuan, Taiwan

**Keywords:** CFSE, [^3^H]-thymidine, Ki-67, lymphocyte proliferation, primary immunodeficiency diseases, proliferation index, stimulation index

## Abstract

**Background:**

Patients with T cell deficiency <10% of normal proliferation are indicated to receive immune reconstruction by hematopoietic stem cell transplantation (HSCT). This study aimed to investigate whether non-radioactive assays can be used to quantitatively detect the lymphocyte proliferation <10% of normal as radioactive [^3^H]-thymidine.”

**Methods:**

Radioactive [^3^H]-thymidine, non-radioactive carboxyfluorescein diacetate succinimidyl ester (CFSE), and Ki-67 protein expressions were used to measure the lymphocyte proliferation as calculated using the stimulation index (SI), subtraction percentage, and proliferation index (FlowJo software). Normal references were established for comparison in the absence of parallel healthy controls.

**Results:**

Normal ranges of mitogen-stimulated lymphocyte proliferation were established as a SI of 15–267 (CSFE 47–92%, Ki-67 42–79%) with phytohemagglutinin (PHA) 5 μg/ml stimulation; 19–139 (CFSE 62–83%, 45–74% Ki-67) with concanavalin-A (ConA) 5 μg/ml stimulation; 7–53 (CFSE 6–23%, Ki-67 10–24%) with pokeweed mitogen (PWM) 0.1 ug/ml stimulation; 3–28 (CFSE 4–10%, Ki-67 5–14%) with candida 10 ug/ml stimulation; and 2–27 (CFSE 6–41%, Ki-67 6–30%) with bacille Calmette-Guerin (BCG) 0.02 ng/ml stimulation. The normalized CFSE-proliferation index was between 2.1 and 3.0. Although there was no significant correlation between these three assays in the healthy controls, the SI value for <10% [^3^H]-thymidine proliferation in those with T cell deficiency was compatible with CFSE- and Ki-67-stained lymphocyte percentages, and validated in patients with *IL2RG, RAG1*, and *ZAP70* mutations. When calculating [^3^H]-thymidine <10% of normal lymphocyte proliferation, the threshold of parallel controls was more reliable than previously established normal references.

**Conclusion:**

The large quantitative value of radioactive [^3^H]-thymidine was more easily recognizable than that for non-radioactive CFSE and Ki-67. Even though the correlation was not significant, those identified to have <10% of normal proliferation by [^3^H]-thymidine could be consistently detected by CFSE and Ki-67, and consequently indicated for HSCT.

## Key Messages

Lymphocyte proliferation evaluated by [^3^H]-thymidine assay and presented as the stimulation index (SI) was similar to the percentages of CSFE and Ki-67-positive staining for lymphocytes, although the correlation was not statistically significant.

As well as opportunistic infections, those with <10% lymphocyte proliferation detected by [^3^H]-thymidine had consistently extremely low (<10%) non-radioactive CFSE and Ki-67-stained lymphocyte percentages compared with their parallel controls and met the indication for hematopoietic stem cell transplantation.

The CSFE proliferation index calculated by FlowJo software consistently showed a value of 1 (normal index range between 2 and 3) in those with <10% lymphocyte proliferation as detected by [^3^H]-thymidine.

## Introduction

Lymphocyte proliferation function is used to assess the increases in proliferation during co-culture with mitogens (e.g., phytohemagglutinin, PHA) and antigens (e.g., candida) (1). Traditionally, a gold standard assay incorporates the radioactive nucleotide [^3^H]-thymidine into DNA, and then the number of mitotic cells with stimulation is compared with those without stimulation (2). To prevent radioactive exposure, the two alternative non-radioactive approaches of the dissociation of carboxyfluorescein diacetate succinimidyl ester (CFSE) (1, 2) and nuclear protein Ki-67 staining (3) have been modified to assess the lymphocyte proliferation as vaccine immune-response and tumor transformation.

Theoretically, non-fluorescent CFSE passively diffuses into cells and is converted into highly fluorescent CFSE when the acetate groups are cleaved by intracellular esterases, subsequently releasing the succinimidyl ester group that reacts with intracellular amines to form fluorescent conjugates by a 2-fold decrease in cellular mean fluorescence intensity (MFI) (1, 2). Ki-67 is a nuclear protein that regulates the cell division and extensively involves in tumor cell proliferation (4–6). The intracellular expression of Ki-67 in cell culture can be used to measure specific T cell responses induced by vaccinations (7–11). Both CFSE and Ki-67 have the potential to replace [^3^H]-thymidine for the assessment of lymphocyte proliferation and reduce exposure to radiation.

Patients with lymphocyte proliferation <10% of normal healthy controls are defined as having profound T cell deficiency, considered to be candidates for hematopoietic stem cell transplantation (HSCT) to reconstruct immunity and prevent them from life-threatening infections. In this study, we first established normal references and then investigated whether radioactive [^3^H]-thymidine was correlated with non-radioactive CFSE and Ki-67 assays with regards to lymphocyte proliferation. We then used these assays to investigate patients with profound T cell deficiency, speculated to what degree their lymphocyte proliferation was impaired, and assessed whether they met the indication for HSCT to reconstruct immunity.

## Materials and Methods

### Subjects

This study was approved by the Institutional Review Board of Chang Gung Memorial Hospital Committee, and informed consent was obtained from the healthy controls (IRB: 201902037A3 and 202001665A3). All methods were performed in accordance with the relevant guidelines and regulations. Venous blood samples (10 ml) from the healthy controls were sent to our laboratory within 48 h. Three classic patients with (severe) combined T and B immunodeficiency (SCID or CID) and identified genetic defects were used as the adjusted references of <10% lymphocyte proliferation.

### The Assessment of Lymphocyte Proliferation by [^3^H]-Thymidine, CFSE, and Ki-67

Lymphocyte proliferation was assessed *in vitro* by 3 days of mitogen (PHA, concanavalin-A [ConA] and/or pokeweed mitogen [PWM]) and/or 7 days of antigen (*Candida albicans* or bacille Calmette-Guerin [BCG] vaccine) stimulation after incubation with [^3^H]-thymidine as previously described (12). The value of [^3^H]-thymidine stimulation index (SI) was obtained from the formula: [stimulated count per minute (c.p.m.) – unstimulated or blank c.p.m.]/unstimulated c.p.m. × 100. A stock solution of CFSE (Molecular Probes, Eugene, OR, USA) was prepared by dissolving CFSE in the DMSO at a concentration of 5 mmol/L. During the labeling process, 1 ml cell suspension was added to the bottom of a falcon tube, followed by the addition of 1:10 PBS-diluted CFSE stock solution. A final CFSE concentration of 5 μmol/L was loaded if the cell count was 2 × 10^6^ cells/ml. After labeling, peripheral blood mononuclear cells (PBMCs) were placed in 96-well round-bottom plates with culture media under the indicated conditions, fixed with 2% paraformaldehyde (Marcon Fine, Rochester, NY), and then analyzed using a BD LSRII flow cytometer (BD Biosciences, San Jose, CA). In addition, the proliferation index of mitotic cells was calculated using the ModFit cell cycle analysis software (FlowJo).

We measured the kinetics of Ki-67 expression in lymphocytes using 10^6^/ml PBMCs in the 96 round tube wells (Sigma, Switzerland, Europe) cultured with blanks, mitogens, and antigens under the indicated conditions. The PBMCs in each well were then fixed, permeabilized (BD Biosciences), and stained with Ki-67 antibodies for 30 min (BD Biosciences). We determined the percentage of lymphocyte proliferation by subtracting those with stimulants and those with blanks only.

### Statistics

Those with <10% of the normal range (an indication for HSCT) were defined by subtraction percentage and mean fluorescent intensity (MFI) from flow cytometry. Correlations between the different analysis methods were assessed using SPSS software version 17 (2009, Chicago, IL). Statistical significance was set at *p* < 0.05.

## Results

### Lymphocyte Proliferation by [^3^H]-Thymidine, CFSE, and Ki-67 in the Healthy Controls

**Figure 1:**
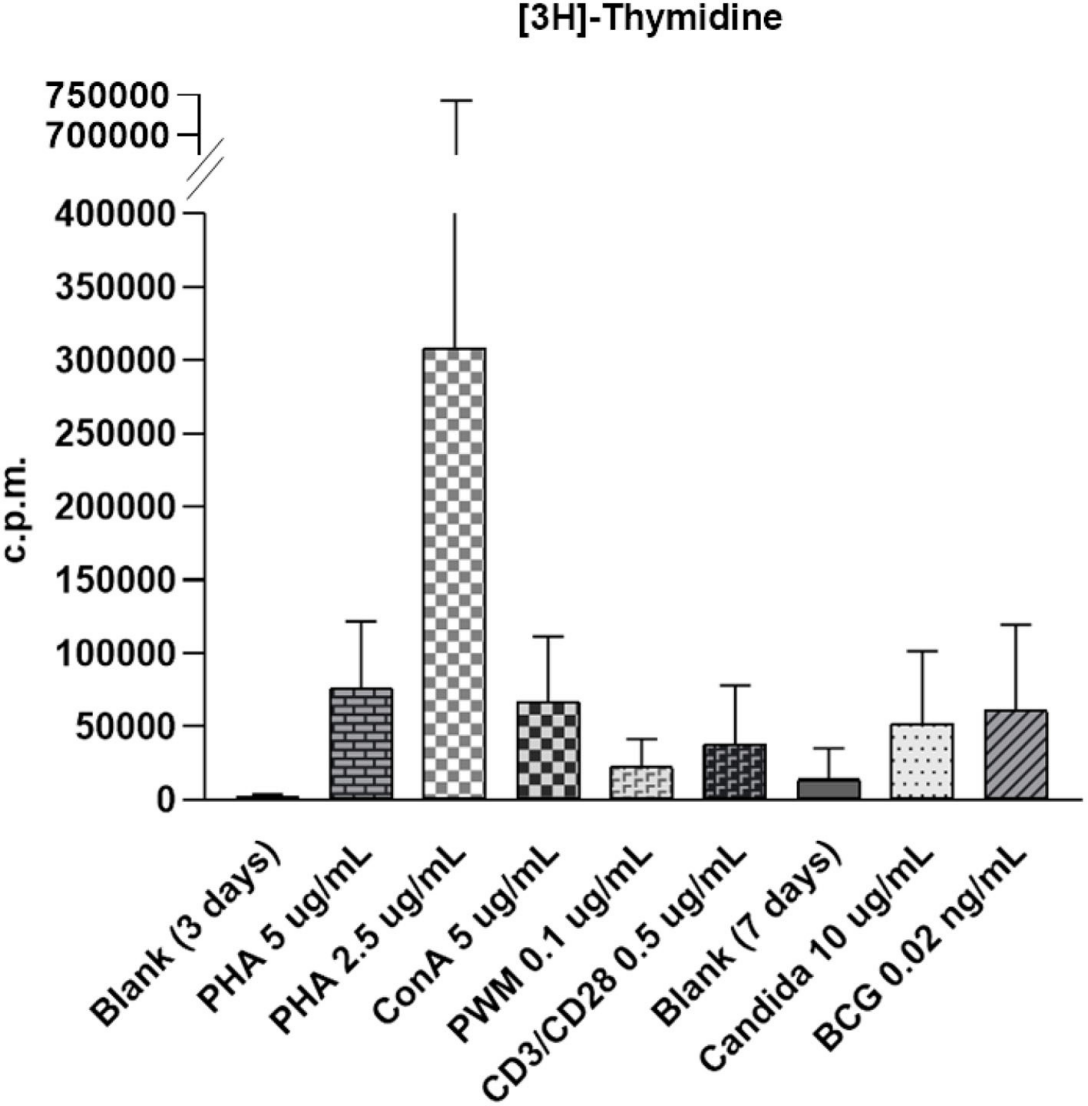
The wide distribution of raw count per minute (c.p.m.) of [^3^H]-thymidine incorporation in stimulated lymphocytes from 29,749 to 136,770 with phytohemagglutinin (PHA) stimulation (5 μg/ml); 27,850 to 1638,459 with PHA stimulation (2.5 μg/ml); 60,802 to 140,756 with concanavalin-A (ConA) stimulation (5 μg/ml); 12,708 to 40,474 with pokeweed mitogen (PWM) stimulation (0.1 μg/ml); 4,232 to 74,700 with CD3/CD28 stimulation (0.5 μg/ml); 22,619 to 122,221 with candida stimulation (0.1 μg/ml), and 10,205 to 146,730 with bacille Calmette-Guerin (BCG) stimulation (0.02 ng/ml). The proliferation index for carboxyfluorescein diacetate succinimidyl ester (CSFE) staining was normalized between 2.1 and 3.0. The [^3^H]-thymidine incorporation in unstimulated lymphocytes in blank was from 148 to 525 in 3-day culture (for mitogen comparison) and from 1,455 to 7,257 in 7 days culture (for antigen comparison).

The healthy controls received stimulation with the mitogens PHA (5 and 2.5 ug/ml), ConA (5 ug/ml), and PWM (0.1 ug/ml), and the antigens candida (10 ug/ml) and BCG (0.02 ng/ml) to assess lymphocyte proliferation. Radioactive [^3^H]-thymidine incorporation, SI, non-radioactive intracellular CFSE, Ki-67 percentage, and MFI were calculated (Supplementary Table 1) to establish the normal ranges in 9–19 healthy controls. The raw c.p.m. of [^3^H]-thymidine in the stimulated lymphocytes showed a wide distribution from 29,749 to 136,770 (Figure 1 and Supplementary Figure 1). In contrast to the left shift with CFSE staining and MFI weaning in each mitosis in the histogram, the value of Ki-67 staining tended to increase and shift to the right in the histogram after stimulation. To narrow this wide range (Supplementary Figure 1) for easier comparisons, we focused on [^3^H]-thymidine SI (by PHA stimulation) instead of using the original raw c.p.m. data to correlate the percentages and MFIs of CFSE- and Ki-67-stained lymphocytes and CFSE-proliferation index (FlowJo software) in the five age-matched healthy controls (Table 1). Regression analysis of the three methods revealed no significant correlation.

**Table 1:** The correlation of mitogen- and antigen-stimulated lymphocyte proliferation as detected by [^3^H]-thymidine, carboxyfluorescein diacetate succinimidyl ester (CFSE), and Ki-67 assays.

**Healthy controls**	**PHA 5 ug/ml**						**PHA 2.5 ug/ml**						
	**Thymidine stimulation index**	**CFSE**		**CFSE proliferation index**	**Ki-67**		**Stimulation index**	**CFSE**		**CFSE proliferation index**	**Ki-67**		
		**%**	**MFI**		**%**	**MFI**		**%**	**MFI**		**%**	**MFI**	
C1	59.6	72.5	3414.2	3.0	79.0	2706.2	37.5	71.4	4182.3	3.0	65.6	2056.1
C2	54.1	89.9	1648.4	3.1	73.1	3929.1	60.9	85.5	1722.8	2.4	41.3	2671.5
C3	216.6	85.5	2532.0	2.9	44.5	4283.5	191.3	82.1	2490.4	2.1	52.4	3957.3
C4	81.2	86.8	2735.8	2.2	56.1	3282.3	99.9	84.1	2405.7	2.2	54.2	3452.1
C5	38.7	81.4	3286.3	2.2	42.2	3737.4	34.9	77.6	3143.1	2.3	31.3	3549.4
*P*-value^a^		*0.717*	*0.779*	*0.680*	*0.470*	*0.335*		*0.438*	*0.508*	*0.213*	*0.731*	*0.211*
	**Con A 5 ug/ml**						**PWM 0.1 ug/ml**					
C1	49.6	64.9	3324.2	2.2	70.8	4270.2	10.4	3.7	8252.4	2.3	19.1	2217.3
C2	38.9	64.2	1767.3	2.1	71.0	4038.4	14.7	8.7	2471.3	1.8	7.7	3947.2
C3	134.7	70.7	2819.2	2.0	53.7	4919.1	53.0	20.9	3849.4	2.0	20.6	6555.1
C4	96.7	80.2	2054.4	2.2	57.1	3273.7	21.5	18.5	2728.1	1.9	22.1	5441.3
C5	30.3	59.1	3821.2	2.0	42.3	2992.2	11.5	11.8	4782.8	1.5	19.2	5698.1
*P*-value^a^		*0.166*	*0.686*	*0.866*	*0.798*	*0.355*		*0.113*	*0.615*	*0.792*	*0.605*	*0.219*
	**Candida 10 ug/ml**						**BCG 1:640 (or 0.02 ng/ml)**					
C1	20.4	2.0	8672.1	1.7	10.3	1941.2	9.2	3.6	9568.3	1.2	8.0	657.5
C2	7.5	3.4	1747.4	1.8	2.1	2453.1	24.6	21.6	1204.1	3.0	10.6	2198.1
C3	14.2	5.5	2689.2	1.4	10.7	5277.4	19.9	15.4	1685.4	2.5	10.1	3681.3
C4	22.2	8.5	1457.8	1.6	9.9	4600.2	26.3	34.5	632.3	2.5	28.2	3002.3
C5	14.1	6.5	1720.5	1.5	11.7	4911.3	11.6	38.2	690.2	2.7	3.1	4015.6
*P*-value^a^		*0.609*	*0.491*	*0.757*	*0.206*	*0.875*		*0.579*	*0.232*	*0.239*	*0.159*	*0.675*

a *The correlation between PHA-stimulated lymphocyte proliferation using [^3^H]-thymidine (as the standard as log transformed or not) and CFSE or Ki-67 was analyzed by the regression analysis and revealed non-significant results (all p > 0.05). There was non-significant correlation between CFSE and Ki-67 assessment. Italics values represent statistic calculations*.

### Impaired Lymphocyte Proliferation in Patients With T Cell Deficiency

**Figure 2:**
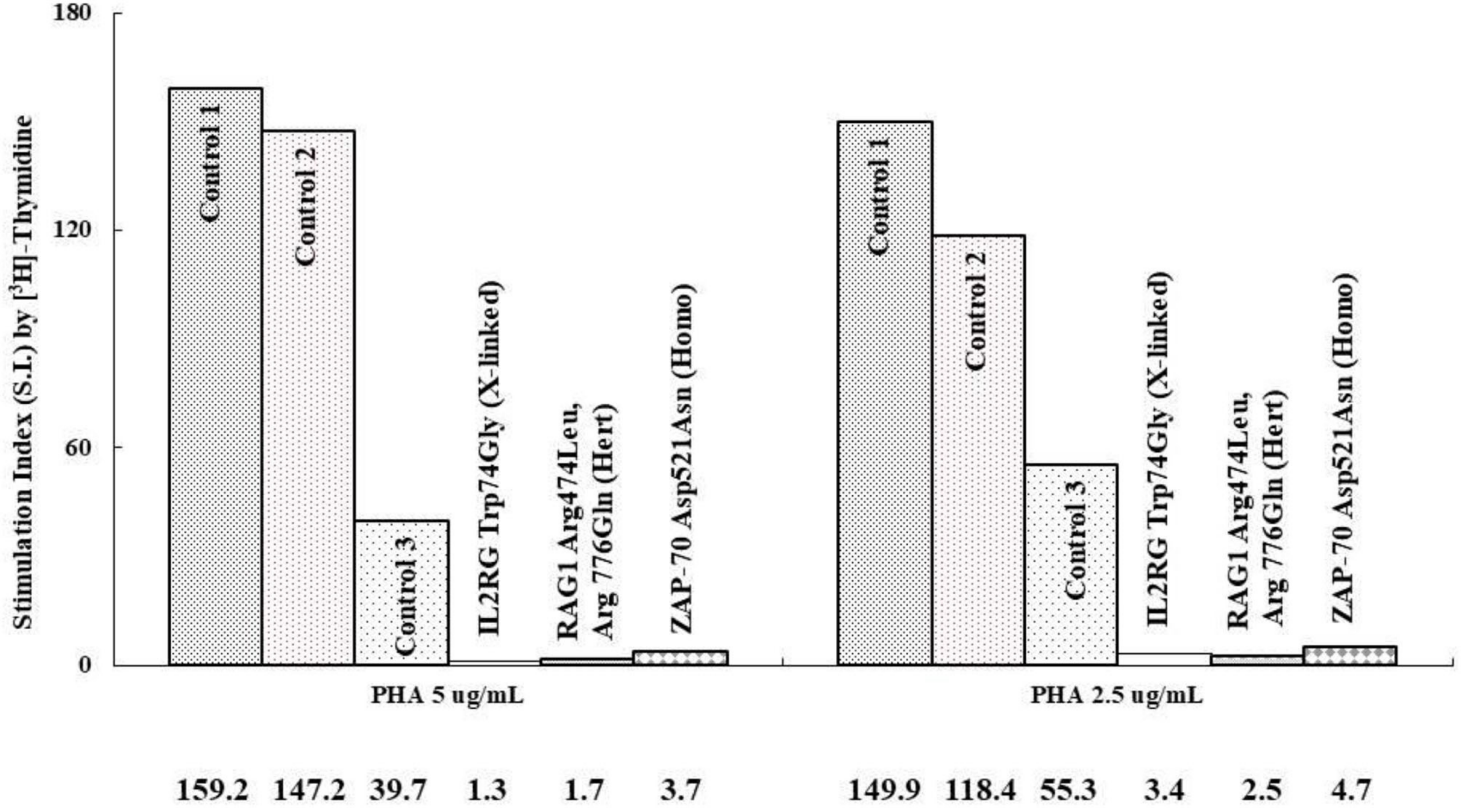
We compared those with profound T cell immunodeficiency causing combined T and B immunodeficiency to the parallel controls (IL2RG mutation with 2.2% CD4, 0.3% CD8, and 38 mm^3^/CD3 vs. Control 1; RAG1 mutation with 3.2% CD4, 0.4% CD8, and 54 mm^3^/CD3 vs. Control 2, and ZAP70 mutation with 46% CD4, 5% CD8, and 1,645 mm^3^/CD3 vs. Control 3) at different time periods. The stimulation index (SI) (stimulated/blank [^3^H]-thymidine incorporation) is shown in the region-axis labels.

**Figure 3:**
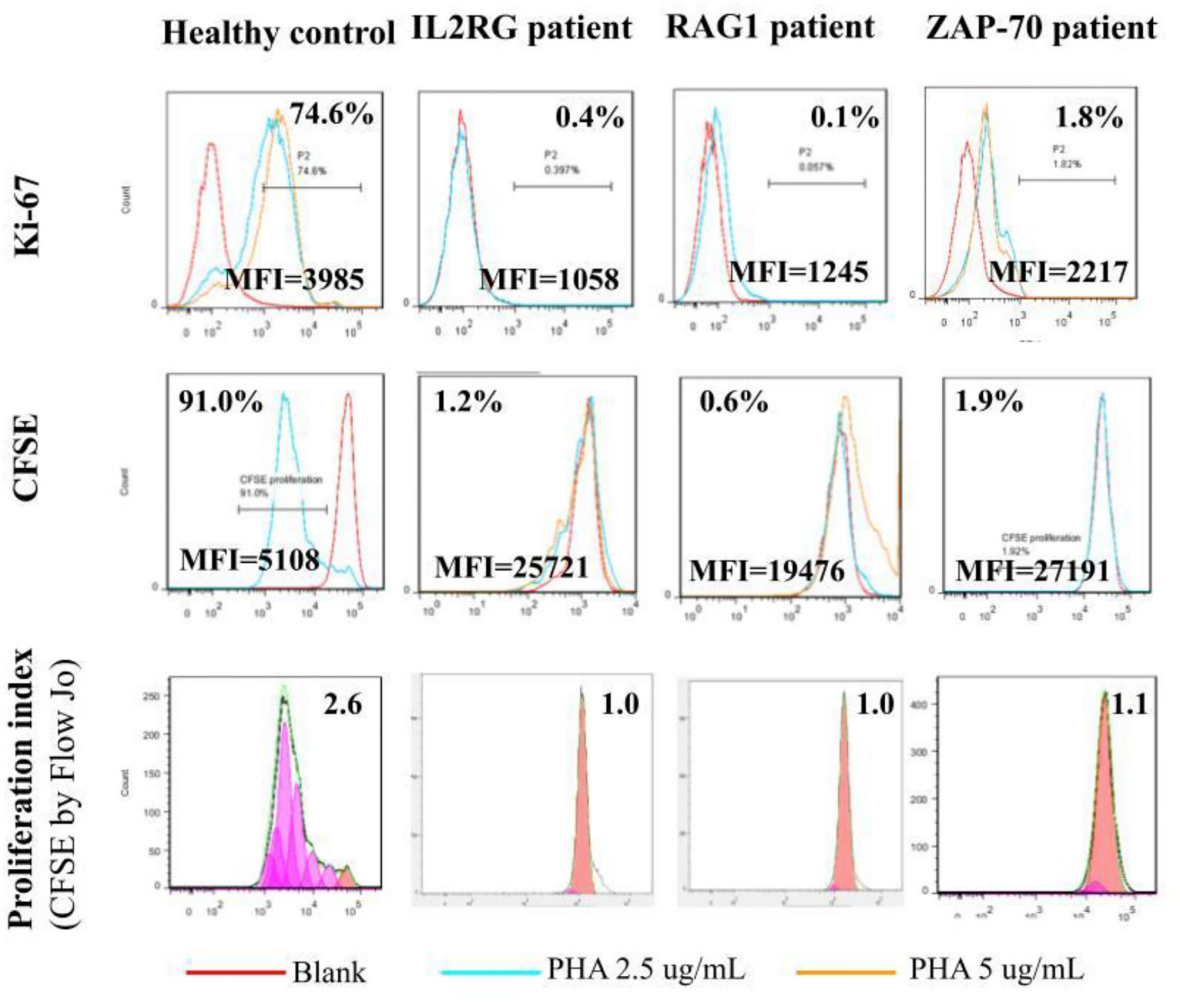
The lymphocyte proliferation assessed by [^3^H]-thymidine incorporation in the patients with the *IL2RG, RAG1*, and *ZAP70* mutation (Figure 2) was <10% of the healthy normal proliferation and consistent with intracellular Ki-67- (0.4, 0.1, and 1.8% vs. 74.6%) and CFSE-stained (1.2, 0.6, and 1.9% vs. 91.0%) lymphocytes. The CFSE proliferation index was approximate 1.0 and far from 2.6 in the healthy control.

Absolute lymphocyte CD3 T cell counts <300/μl and <10% of normal proliferation to the PHA stimulation are indications for HSCT in patients with profound T cell deficiency who are presented with (severe) combined T and B cell immunodeficiency (SCID or CID). Thus, we defined a threshold value <10% of normal proliferation from the healthy controls (Supplementary Table 1) as a potential indication for HSCT. We simultaneously assessed both index cases and parallel healthy controls while evaluating PHA-stimulated lymphocyte proliferation. For those with *IL2RG* [Trp74Gly; X-linked; their immunophenotyping was 2.2% CD4, 0.3% CD8, and 38/μl CD3], *RAG1* [Arg474Leu, Arg776Gln; heterozygous; 3.2% CD4, 0.4% CD8, and 54/μl CD3], and *ZAP70* [Asp521Asn; homozygous; 46% CD4, 5% CD8, and 1645/μl CD3] mutations, which are responsible for T cell deficiency, their PHA-stimulated lymphocyte proliferation as assessed by [^3^H]-thymidine incorporation was <10% compared with their parallel controls (Figure 2), which was compatible with the percentages of CFSE and Ki-67 positive-stained lymphocytes, and consistent with the proliferation index from CFSE staining (Figure 3). The accompanying blood sample of the control C3 showed <10% PHA-stimulated lymphocyte proliferation [39.7; 55.3 in Control 3 in Figure 2]. In addition, we assessed the patient and blood sample control C3 using the other stimulations (mitogens with ConA, PWM, and CD3/CD28, and antigens with candida and BCG) incorporating [^3^H]-thymidine, and the results showed consistently low values (data not shown) supporting the indication for immune reconstruction by suitable HSCT, as our report with a good prognosis to date (14).

## Discussion

Lymphocyte proliferation is a clinical screening method used to evaluate the lymphocyte response of a patient to mitogens and antigens *in vitro*. If defective molecules involving activation pathways inhibit [^3^H]-thymidine nucleotide incorporation, incremental CFSE dilution, and nuclear Ki-67 entrance, the quantitative value of [^3^H]-thymidine measured using a beta-counter and flowcytometric percentages of CFSE and Ki-67 will increase less during stimulation, and this can explain recurrent opportunistic infections. In this study, we established standard normal ranges for comparison without the accompanying controls. The results showed that the quantitative value obtained with [^3^H]-thymidine incorporation was more sensitive and easily recognizable than that of CFSE and Ki-67 staining by flowcytometry, which depends more on skilled operators.

Phytohemagglutinin-induced lymphocyte proliferation <10% in patients with severe T cell deficiency indicates HSCT for immune reconstruction. Otherwise, they will have increased susceptibility to opportunistic infections and die in infancy. Neonatal screening for T cell receptor excision circle (TREC) in the Guthrie card and consequently functional lymphocyte proliferation can identify patients with profound T cell immunodeficiency early (13) before suffering from life-threatening critical infections. Acceptable lymphocyte proliferation function can almost totally exclude patients with profound T cell defects despite a low TREC value in their Guthrie cards, which could be due to maternal immunosuppressive factors, prematurity, sepsis, Down's syndrome, congenital leukemia, and gastrointestinal and chromosome anomalies.

Our results indicate that [^3^H]-thymidine incorporation to detect lymphocyte proliferation is more sensitive and recognizable (Figure 2) than flowcytometric intracellular CFSE and Ki-67 staining (Figure 3). Even though a non-significant correlation existed among these three methods, those with extreme T cell deficiency of <10% could be detected by [^3^H]-thymidine, and the percentage but not MFI could be detected by CFSE and Ki-67-stained lymphocytes. The CFSE proliferation index of PHA-stimulated lymphocyte proliferation also showed consistent fixed-style histograms (~1 vs. 2–3 in normal controls) without any detectable proliferation.

Using advanced molecular techniques, such as whole-exome and genome sequencing, the genetic defects of primary immunodeficiency disorders (or inborn errors of immunity) can be identified in ~20% of all those who are screened, and even up to 40% when including a traditional candidate genetic approach (15, 16). Most patients with predominant antibody deficiency have common variable immunodeficiency diseases (CVID), and they often have a relatively better prognosis under regular immunoglobulin treatment. Approximately 10% of patients with CVID have identifiable genetic defects, and it is possible to predict their clinical course and outcomes. However, some CVID patients with unknown genetic defects gradually develop severe T cell deficiency with increasing age, and consequently suffer from opportunistic infections (17, 18). In this situation, assessing lymphocyte proliferation using one of these three methods can allow physicians to decide in a timely manner whether to consider opportunistic prophylactics and even HSCT if the value of PHA-lymphocyte proliferation is <10%.

This study should be interpreted in light of its limitations. First, the three assays quantify lymphocyte proliferation using different mechanisms: (1) by incorporating a nucleotide ([^3^H]-thymidine); (2) by binding vital division-protein (Ki-67); and (3) by decreasing the concentration by 2-fold during each mitosis (CFSE). Therefore, the expressions of these three biomarkers could vary in the different stages of lymphocyte proliferation which may explain the non-significant correlation with each other. Second, the optimal conditions for the three biomarkers to quantify the strongest expression were different. Time-sequence analysis may reveal the adequate time-point to harvest. Third, those with <10% lymphocyte proliferation may have been misclassified as having >10% normal lymphocyte proliferation according to the previously established normal range in which the tested samples were obtained without parallel controls. Fourth, large-scale and time-sequence analysis studies are needed to investigate whether non-radioactive assays are correlated with radioactive lymphocyte proliferation, especially those without the profound impairment of lymphocyte proliferation.

In conclusion, those with <10% lymphocyte proliferation detected by radioactive [^3^H]-thymidine who met the indication for HSCT had similarly extremely low non-radioactive CFSE- and Ki-67-stained lymphocyte percentages compared with their parallel controls rather than the previously established normal range. Despite a non-significant correlation between these three assays, the large quantitative value with [^3^H]-thymidine is easily recognizable, whereas CFSE and Ki-67 staining depend on skilled operators for flowcytometric gating and subtraction. Of course, the clinical clues of opportunistic infections and autoimmune disorders should never be neglected for effective management.

## Data Availability Statement

The original contributions presented in the study are included in the article/Supplementary Material, further inquiries can be directed to the corresponding author/s.

## Ethics Statement

The studies involving human participants were reviewed and approved by the Institutional Review Board of our Chang Gung Memorial Hospital Committee. Written informed consent to participate in this study was provided by the participants' legal guardian/next of kin.

## Author Contributions

H-JL, C-CG, and W-IL carried out the molecular genetic studies, participated in the sequence alignment, and drafted the manuscript. W-FL, H-YY, C-JL, and S-HF carried out the immune function. W-IL and J-LH participated in the design of the study and performed the statistical analysis. S-HC participated in the study to care for critical patients. W-IL conceived the study, participated in its design and coordination, and helped to draft the manuscript. All authors read and approved the final manuscript.

## Funding

This work was supported by the Chang-Gung Medical Research Progress (Grants CMRPG3K2231, CMRPG3K2232, and CMRPG3M0351), the National Science Council (Grants NMRPG3K0361 and 3L0241; MOST 109-2314-B-182A), and Taiwan Foundation for Rare Disorders (TFRD).

## Conflict of Interest

The authors declare that the research was conducted in the absence of any commercial or financial relationships that could be construed as a potential conflict of interest.

## Publisher's Note

All claims expressed in this article are solely those of the authors and do not necessarily represent those of their affiliated organizations, or those of the publisher, the editors and the reviewers. Any product that may be evaluated in this article, or claim that may be made by its manufacturer, is not guaranteed or endorsed by the publisher.

## Supplementary Material

Supplementary Figure 1 Under stimulation with the indicated mitogens and antigens, the normal values of lymphocyte proliferation were evaluated by [^3^H]-thymidine stimulation index (SI), carboxyfluorescein diacetate succinimidyl ester (CFSE)- or Ki-67-stained percentages, CFSE or Ki-67 mean fluorescent intensity (MFI), and CFSE proliferation index.

Supplementary Table 1 The range of PHA-stimulated lymphocyte proliferation as detected by [^3^H]-thymidine, CFSE and Ki-67 assays in the normal healthy controls.
