# Supplementary figures and images for: Comparison of [3H]-Thymidine, Carboxyfluorescein Diacetate Succinimidyl Ester and Ki-67 in Lymphocyte Proliferation
Source: Front Pediatr. 2022 Apr 25;10:638549. doi: 10.3389/fped.2022.638549 (PMC9082031; doi:10.3389/fped.2022.638549)

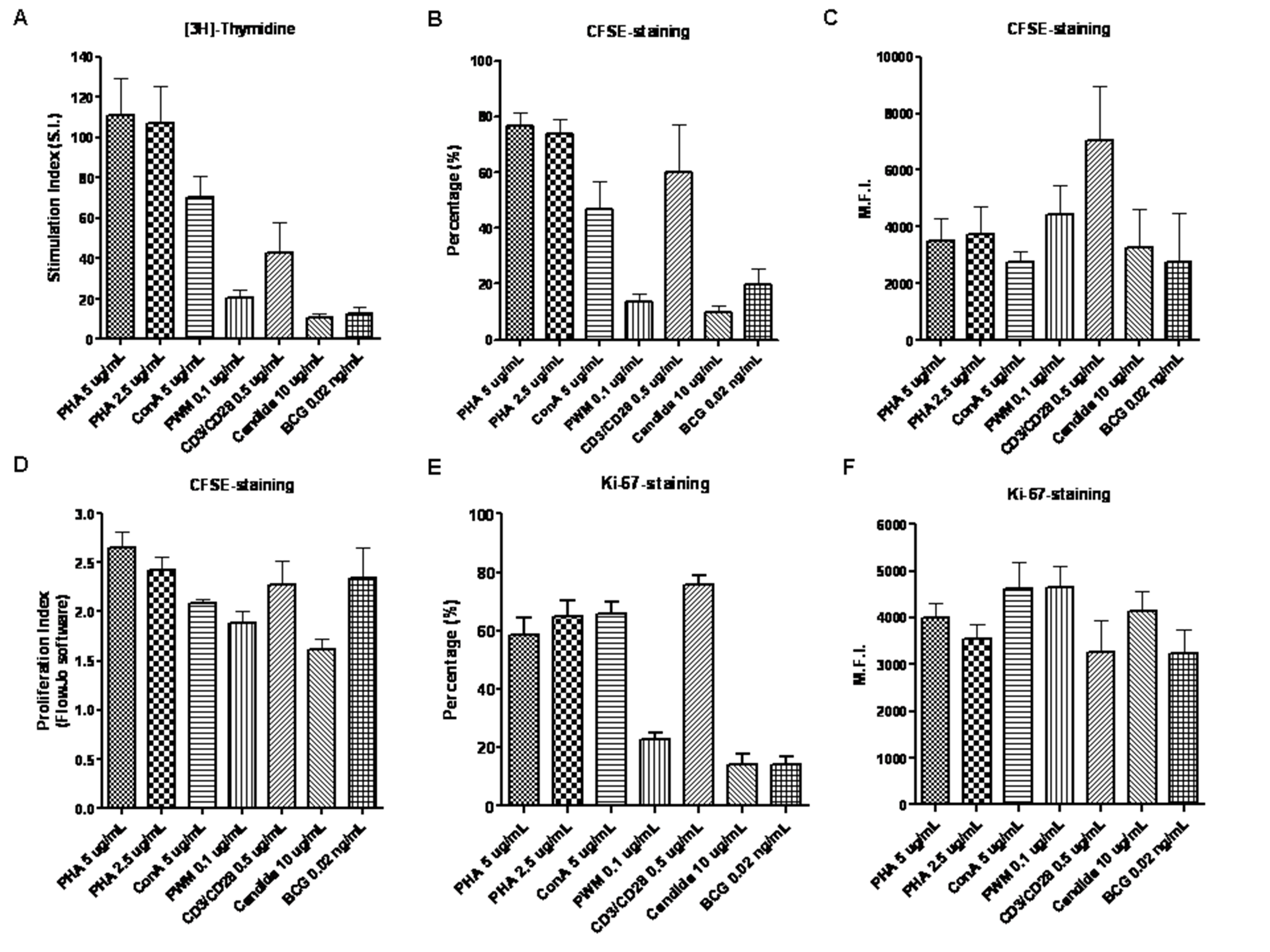

Supplement: Supplementary Figure 1 — Under stimulation with the indicated mitogens and antigens, the normal values of lymphocyte proliferation were evaluated by [3H]-thymidine stimulation index (SI), carboxyfluorescein diacetate succinimidyl ester (CFSE)- or Ki-67-stained percentages, CFSE or Ki-67 mean fluorescent intensity (MFI), and CFSE proliferation index. [file Image_1.TIF]
