# Supplementary material for: Comparison of [3H]-Thymidine, Carboxyfluorescein Diacetate Succinimidyl Ester and Ki-67 in Lymphocyte Proliferation
Source: Front Pediatr. 2022 Apr 25;10:638549. doi: 10.3389/fped.2022.638549 (PMC9082031; doi:10.3389/fped.2022.638549)
Supplement: Supplementary Table 1 — The range of PHA-stimulated lymphocyte proliferation as detected by [3H]-thymidine, CFSE and Ki-67 assays in the normal healthy controls. [file Table_1.DOCX]

**Supplemental Table 1.** The range of PHA-stimulated lymphocyte proliferation as detected by [^3^H]-thymidine, CFSE and Ki-67 assays in the normal healthy controls

|  | **PHA 5 ug/ml** | | | **PHA 2.5 ug/ml** | | |
| --- | --- | --- | --- | --- | --- | --- |
|  | **Control Number** | **Range** | **Below 10% *** | **Control Number** | **Range** | **Below 10% *** |
| **[^3^H ]-thymidine** | 18 |  |  | 18 |  |  |
| c.p.m. |  | 29749-136770 | 2974.9 |  | 27850-114529 | 2785.0 |
| Stimulation index |  | 14.9-266.6 | 1.49 |  | 16.0-309.1 | 1.60 |
| **CFSE** | 11 |  |  | 9 |  |  |
| Percentage |  | 46.6-92.1 | 4.66 |  | 41.4-81.7 | 4.14 |
| MFI |  | 3413-34363 | 341.3 |  | 4157-24806 | 415.7 |
| Proliferation index |  | 2.2-3.0 |  |  | 2.1-3.0 |  |
| **Ki-67** | 10 |  |  | 9 |  |  |
| Percentage |  | 42.2-79.0 | 4.22 |  | 31.3-70.1 | 3.13 |
| MFI |  | 2706-5964 | 270.6 |  | 2056-5126 | 205.6 |
|  |  |  |  |  |  |  |

* Without accompanying controls in urgency (neonatal intensive care unit), reference ranges previously calculated in our own laboratory cannot help but take into comparison. However, the borderline threshold of <10% of normal PHA proliferation previously settled-up in our own laboratory is possibly lower than the accompanying controls if available. To avoid this issue (delay to receive HSCT), we suggest perform lymphocyte-proliferation with parallel controls because test- and control-lymphocytes meet the similar shipping-travel duration and conditions.
